# Histological and inflammatory effects of 26.5 GHz quasi-millimeter wave exposure on rat skin

**DOI:** 10.3389/fpubh.2025.1580155

**Published:** 2025-06-16

**Authors:** Etsuko Ijima, Akiko Nagai, Kun Li, Takashi Hikage, Naomi Kamizawa, Emi Hidaka, Yukina Tsuruta, Tatsuya Ishitake, Hiroshi Masuda

**Affiliations:** ^1^Department of Environmental Medicine, Kurume University School of Medicine, Kurume, Japan; ^2^Department of Anatomy, School of Dentistry, Aichi Gakuin University, Nagoya, Japan; ^3^Advanced Wireless and Communication Research Center (AWCC), The University of Electro-Communications, Chofu, Japan; ^4^Faculty of Information Science and Technology, Hokkaido University, Sapporo, Japan

**Keywords:** 5G, quasi-millimeter wave, localized exposure, skin temperature, inflammation

## Abstract

**Introduction:**

Information regarding the biological effects of localized exposure to quasi-millimeter waves (qMMW) is limited. Given that qMMW exposure can elevate skin temperature and potentially induce thermal injury, further investigation is required. In this study, we aimed to evaluate histological changes and the expression of inflammation-related markers in rat skin tissue locally exposed to 26.5 GHz qMMW, as well as investigate the threshold for inflammatory responses.

**Methods:**

The dorsal skin of rats was locally exposed to 26.5 GHz qMMW at absorbed power densities (APD) of 0, 250, 370, and 500 W/m^2^ for 18 min using a patch antenna. Histological changes and expression patterns of inflammation-related markers were examined in skin tissue sections exposed to qMMW. Furthermore, serum levels of prostaglandin E_2_ (PGE_2_), tumor necrosis factor-*α* (TNF-α), and interleukin-6 (IL-6) were measured at each post-exposure time point.

**Results:**

Histological analysis revealed burn-like tissue damage in the 500 W/m^2^ exposure group, characterized by subepidermal blister formation, epidermal thickening, and dermal edema, which increased in severity over time. Conversely, the lower exposure groups (250 and 370 W/m^2^) showed no distinct morphological changes, similar to the sham group. The 500 W/m^2^ group exhibited significantly elevated expression of inducible nitric oxide synthase (iNOS) and ionized calcium-binding adapter molecule 1 (Iba1), particularly in the dermis, dermal white adipose tissue, and sebaceous glands. Serum levels of PGE_2_ increased in a dose-dependent manner at 24 and 72 h; TNF-*α* and IL-6 remained undetectable. The skin temperature increased during qMMW exposure, reaching 39.0 ± 0.6°C, 42.4 ± 0.9°C, and 44.8 ± 1.2°C at APDs of 250, 370, and 500 W/m^2^, respectively.

**Discussion:**

Localized exposure of rat skin to qMMW induces burn-like tissue degeneration and triggers an inflammatory response. This effect may be thermally induced by qMMW irradiation, with the threshold estimated to range between 370 and 500 W/m^2^ APD under the present experimental conditions. Few studies have demonstrated MMW-induced inflammatory responses in the skin. To the best of our knowledge, this is the first study to clearly define the threshold using APD as a reference. These findings may contribute useful evidence for future revisions of exposure guidelines.

## 1 Introduction

When the body is exposed to high-intensity millimeter waves (MMWs; 20–300 GHz), including the quasi-millimeter wave band (qMMWs; ≥20 GHz and <30 GHz), the body surface primarily absorbs radiofrequency energy, resulting in heat generation and an increase in skin temperature. Due to their short wavelengths, the penetration depth of MMWs into biological tissues is limited to a few millimeters, with most absorbed energy confined to the skin tissue (1). This absorbed energy is converted into heat, which triggers an elevation in skin temperature. Increasing temperature results in physiological responses such as sweating and enhanced blood flow, and when the temperature surpasses a critical threshold, there is a risk of thermal injury. The skin, comprising approximately 16% of the total body weight (2), is the largest organ of the human body and plays crucial roles not only in protection from external factors and thermoregulation but also in immune function contributing to homeostasis maintenance (3). Therefore, evaluating the effects of MMW exposure on skin tissue in terms of temperature changes is essential to ensure the safe implementation of 5G technology.

Although several studies have investigated the effects of MMW exposure on skin, *in vivo* data remain limited. For instance, Millenbaugh et al. (4) exposed rat skin to 35 GHz MMWs and analyzed their effects. Exposure to an incident power density of 750 W/m^2^ increased skin temperature, with collagen and skeletal muscle fiber degeneration and adipocyte lysis observed 24 h post-exposure. However, the authors did not provide detailed information regarding the relationship between the exposure dose and skin temperature or the threshold of exposure required to induce tissue degeneration. Similarly, Parker et al. conducted a localized exposure study on pig skin using 95 GHz MMWs to evaluate temperature changes and histological changes. However, due to the extremely high exposure intensity, the relationship between mild exposure intensity and tissue degeneration could not be established (5). Although these studies provided insights into MMW-induced skin effects, they failed to establish a clear relationship among exposure intensity, temperature elevation, and histological alterations, making it necessary to strengthen the evidence to discuss skin damage thresholds. Furthermore, the international organizations’ guidelines for the safe use of MMW (6, 7) highlight that biological evidence is still limited and emphasize the need for additional experimental research.

Based on this background, we conducted a preliminary study as a first step toward further elucidating the effects of MMW exposure on the skin. Specifically, qMMW at 26.5 GHz were locally applied to the dorsal skin of rats, and the relationship between exposure intensity and skin temperature was investigated (8). The results demonstrated that skin temperature increased almost linearly with absorbed power density (APD), and that exposure at 250 W/m^2^ for 6 min raised the skin temperature by approximately 5°C, reaching an actual temperature of around 39°C. These findings largely support the biological rationale underlying the revised 2020 guidelines published by the International Commission on Non-Ionizing Radiation Protection (ICNIRP) (6).

In accordance with these guidelines, exposure resulting in a local tissue temperature exceeding 41°C is considered potentially harmful. Adopting a conservative approach, the guidelines set an operational adverse health effect threshold for superficial tissues, such as skin and subcutaneous fat (classified as Type 1 tissues), at a 5°C rise above normothermia under radiofrequency electromagnetic field exposure. Furthermore, they estimate that an APD of approximately 200 W/m^2^ would be required to induce this temperature increase and set recommended exposure limits at 100 W/m^2^ for occupational settings and 20 W/m^2^ for the general public. Thus, exposure around 250 W/m^2^, which experimentally induced a 5°C rise in skin temperature, may have triggered potential adverse effects in rat skin as defined by the guidelines. However, the prior study only examined temperature data and did not clarify whether histological or cellular changes had occurred in the exposed tissue. Demonstrating not only temperature elevation but also tissue damage under such exposure conditions would provide more robust biological support for the current safety standards.

In the present study, we aimed to determine whether local exposure to 26.5 GHz qMMW induces histological alterations in skin tissue and whether these changes exhibit dose dependence with respect to APD. Specifically, we locally exposed the dorsal skin of rats to qMMW and evaluated histological changes along with the expression of multiple inflammation-related markers. Additionally, to elucidate the threshold for skin tissue alterations, we assessed not only exposure at 250 W/m^2^ but also at 1.5-fold (370 W/m^2^) and 2-fold (500 W/m^2^) higher intensities.

## 2 Materials and methods

### 2.1 Animals

Sixty-one male Sprague–Dawley rats (8–9 weeks old; Japan SLC, Shizuoka, Japan) were used for this experiment. The rats were maintained on a standard pellet diet, with *ad libitum* access to water in a room with a 12-h light/dark cycle at 22.5 ± 1°C and 50 ± 20% relative humidity. The dorsal body hair of rats was shaved before starting the experiment. All experimental procedures were conducted in accordance with the ethical guidelines for animal experiments at Kurume University School of Medicine (approval numbers: 2021–150 and 2022–114).

### 2.2 Exposure to 26.5 GHz qMMW

**Figure 1:**
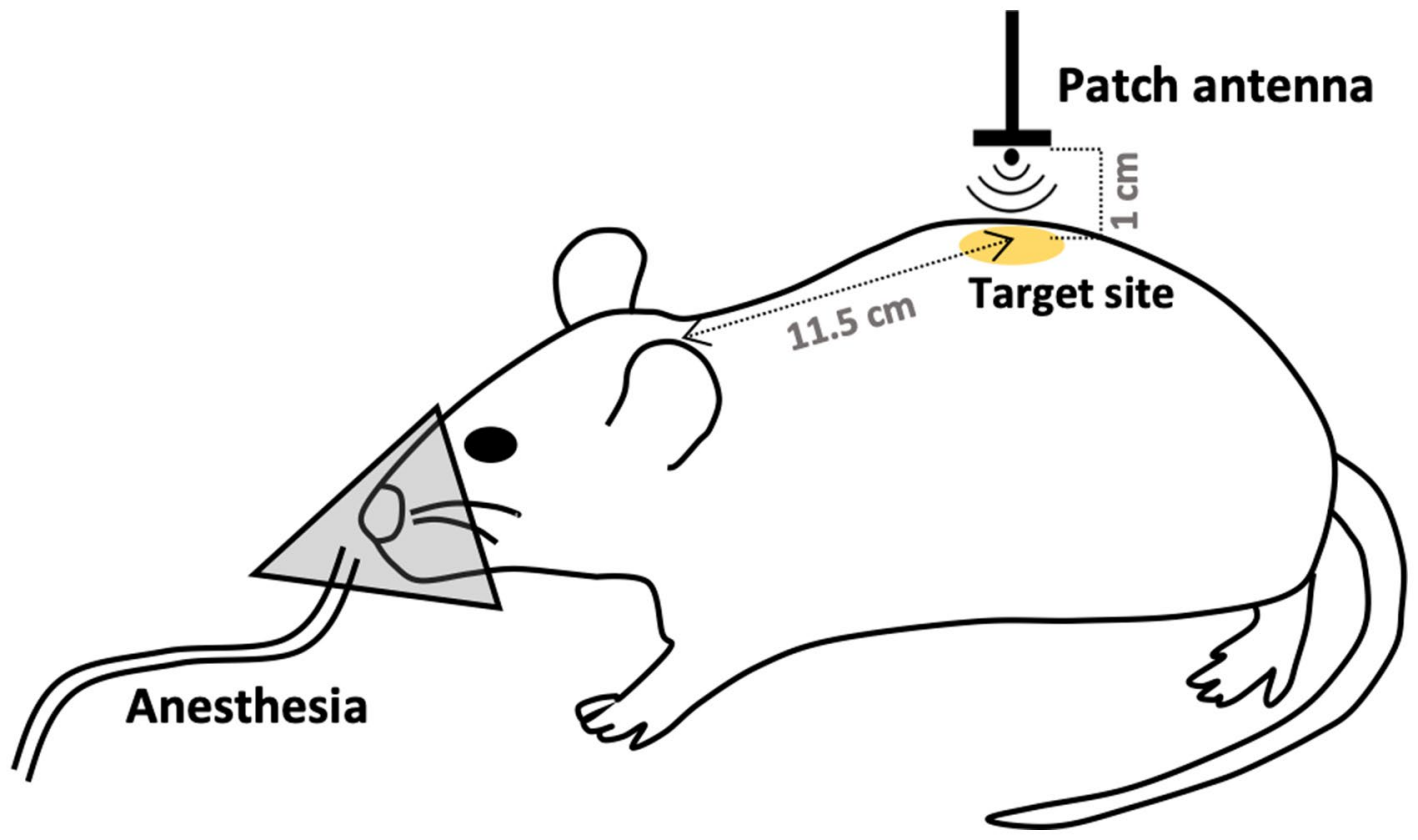
Schematic of the 26.5 GHz qMMW exposure protocol. The dorsal skin of rats was locally exposed to 26.5 GHz quasi-millimeter wave (qMMW) using a custom-designed patch antenna. The target site was defined as 11.5 cm posterior to the base of the ear on the midline. Localized irradiation was applied to an area with a diameter of approximately 20 mm. The amplified signal was emitted from the patch antenna and directed toward the target surface positioned 1 cm in front of the antenna aperture.

Exposure to 26.5 GHz qMMW was performed as described in our previous study (8). The system consisted of a signal generator (MG3692C; Anritsu, Tokyo, Japan), an amplifier (AMP6034-20; Exodus Advanced Communications, Las Vegas, NV), a waveguide, a Y-shaped splitter connected to a power meter (EPM-442A; Hewlett Packard, Palo Alto, CA), a semi-rigid coaxial cable, and a patch antenna optimized for localized exposure. A continuous sinusoidal 26.5 GHz signal was generated by the signal generator, amplified, and transmitted to the patch antenna via a coaxial cable. The amplified signal was emitted from the patch antenna and irradiated the target skin surface located 1 cm in front of the antenna aperture (Figure 1). The input power to the antenna was continuously monitored by a power meter incorporated between the amplifier and the patch antenna.

In this study, the dorsal skin of rats was used as the target for qMMW exposure. The center of the localized irradiation area was defined as a point 11.5 cm posterior to the base of the ears along the midline (Figure 1). Dosimetric analysis for determining the spatially averaged APD was performed under conditions described previously (8), using the antenna input power and a four-layer skin model comprising the epidermis, dermis, dermal white adipose tissue, and panniculus carnosus. Layer thicknesses were based on the mean values from excised tissue data (9), and dielectric properties were obtained from (1). The analysis indicated that the localized exposure area had a diameter of approximately 20 mm, as confirmed by thermographic imaging. The spatially averaged APD was estimated to be 250, 370, and 500 W/m^2^ at antenna input powers of 0.317, 0.476, and 0.63 W, respectively.

All exposures, including those for the sham group, were conducted under 2% isoflurane anesthesia with oxygen for 18 min. The number of rats per group was: sham (*n* = 15), 250 W/m^2^ (*n* = 12), 370 W/m^2^ (*n* = 14), and 500 W/m^2^ (*n* = 17). The exposure duration of 18 min was chosen because, under the current experimental conditions, thermal balance at the body surface of sham-exposed rats could be maintained for approximately 30 min, enabling detection of subtle temperature changes during qMMW exposure (8). Pilot experiments also confirmed that this exposure time was sufficient to induce detectable histological changes. Additionally, using the same exposure duration allowed for direct comparison with previously reported findings from brain tissue exposed to radiofrequency electromagnetic fields (10). In addition to the above groups, a cage control group (*n* = 3) was included. These animals were housed in cages without being subjected to any qMMW exposure, including 0 W/m^2^.

### 2.3 Temperature measurement

Temperatures at the target site during qMMW exposure were measured following the methodology described in our previous study (8). Temperature data were collected using fiber optic thermometers with a 0.5 mm tip diameter (m600; Advanced Energy Industries, Inc., Denver, CO/FL-2400; Anritsu Corporation, Tokyo, Japan) and an A/D converter (PL3516; AD Instruments, Dunedin, New Zealand). The thermometer probe was placed in contact with the dorsal skin at the center of the target site. In this study, the skin temperature analysis was conducted using 53 animals in total, 48 of which had already been included in a previously published study [P1 in (8)].

### 2.4 Histologic assessment

The target skin tissue was histologically analyzed to evaluate the effects of qMMW exposure on skin morphology. At 24 or 72 h post-exposure, euthanasia was carried out under deep anesthesia induced by an intraperitoneal injection of secobarbital (100 mg/kg body weight) and pentazocine (10 mg/kg body weight), followed by exsanguination. A 1 × 1 cm square of skin tissue was excised from the target site and fixed in 10% neutral buffered formalin at 4°C overnight, followed by immersion in 20% sucrose at 4°C for 48 h for cryoprotection. The tissues were embedded in Tissue-Tek O. C. T. Compound (Sakura Finetek, Tokyo, Japan) and frozen in cold isopentane. Cryosections were prepared by cutting 12-μm-thick sections using a cryostat (CM1950OUVV; Leica, Wetzlar, Germany).

To observe morphological changes in the target tissue, cryosections were stained with hematoxylin and eosin (H&E) (Muto Pure Chemicals Co., Ltd., Tokyo, Japan). The severity of histological changes was evaluated based on six criteria. The epidermal thickness was assessed as none (0), partial (1), or widespread (2). Leukocyte infiltration was classified as absent (0), moderate (1), or severe (2). Intercellular dermal edema was categorized as none (0), moderate (1), or severe (2). Collagen degeneration, granulation, and blister formation in the epidermis were scored as absent (0) or present (1).

Four independent researchers examined two sections per animal in a blinded manner using a light microscope (BX51; Olympus, Tokyo, Japan). The mean score of the four researchers was calculated for each rat, and this value was used as the individual score. A total of 5–8 rats per group were used for histological analysis.

### 2.5 Immunohistochemistry

We analyzed the expression levels of two proteins involved in inflammation, inducible nitric oxide synthase (iNOS) and ionized calcium-binding adapter molecule 1 (Iba1). Cryosections were prepared as described above and subjected to immunohistochemical analysis following a standard protocol. The primary antibodies used were iNOS (1:400) (ab178945; Abcam, Cambridge, UK) and Iba1 (1:400) (019–19,741; FUJIFILM Wako Pure Chemical Corporation, Osaka, Japan). The secondary antibody was donkey anti-rabbit IgG H&L conjugated to horseradish peroxidase (1:200) (A0545; Sigma-Aldrich, St. Louis, MO). Staining was performed using the DAB Substrate Kit (SK-4100; Vector Laboratories, Newark, CA).

Following immunostaining, the tissue sections were observed under a light microscope (BX51; Olympus, Tokyo, Japan), and bright-field microscopy images were acquired using a CCD camera (DP74; Olympus, Tokyo, Japan). To evaluate Iba1 expression, images from 5 to 8 rats per group were analyzed using Metamorph software (ver. 7.8.0.0, Molecular Devices, San Jose, CA). The region of interest (ROI) for analysis was limited to the dermal layer and deep dermal white adipose tissue (dWAT), and hair follicles, sebaceous glands, and blood vessels were excluded.

The positive chromogen-stained signals were detected using RGB color detection, and the percentage of the positive area relative to the total ROI area was calculated as the positive area (%). A standardized threshold for RGB levels was established to ensure consistency and eliminate the variability caused by differences in staining procedures. Any apparent false-positive signals were excluded from the analysis.

### 2.6 Enzyme-linked immunosorbent assay (ELISA)

Serum biomarkers were quantified using ELISA. The analysis focused on prostaglandin E_2_ (PGE_2_), tumor necrosis factor-*α* (TNF-α), and interleukin-6 (IL-6), which are involved in inflammatory responses.

Blood samples were collected from the jugular vein immediately before and after exposure. Blood samples were obtained from the abdominal aorta at the time of sacrifice and 24 or 72 h post-exposure. The serum was separated from whole blood and stored at −80°C until analysis.

In each group (*n* = 6–9 rats), serum biomarker levels were measured using specific ELISA kits (PGE_2_: CSB-E07967r, CUSABIO Technology LLC, Houston, TX; IL-6: R6000B, R&D Systems, Inc., Minneapolis, MN; TNF-*α*: EK0526, Boster Bio, Pleasanton, CA) following the manufacturer’s instructions. Measurements were performed using a microplate reader (Multiskan FC Basic; Thermo Fisher Scientific, Waltham, MA).

### 2.7 Statistical analysis

Differences in the effects of qMMW exposure across exposure intensities were statistically evaluated using the Kruskal–Wallis test followed by the Steel test. Statistical analyses were performed using Bell Curve for Excel software (version 4.07; Social Research Information Co., Ltd., Japan). A *p*-value of < 0.05 was considered statistically significant.

## 3 Results

### 3.1 Histological changes in skin tissue

**Figure 2:**
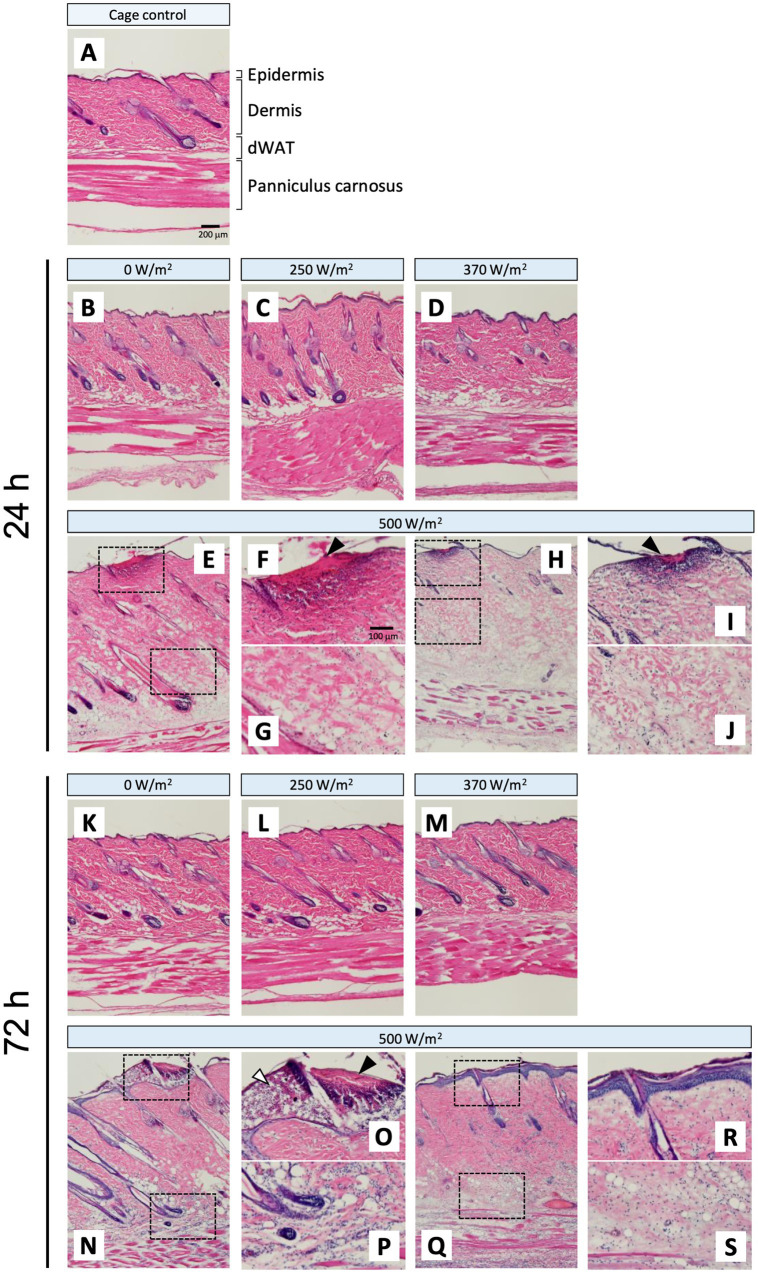
Histopathological changes in rat skin following 26.5 GHz qMMW exposure. Representative hematoxylin and eosin (H&E)-stained images of the target site at 24 h **(B–J)** and 72 h **(K–S)** post-exposure. **(A)** Cage control; **(B,K)** 0 W/m^2^; **(C,L)** 250 W/m^2^; **(D,M)** 370 W/m^2^; **(E–J,N–S)** 500 W/m^2^. High-magnification views **(F,G,I,J,O,P,R,S)** show the dotted rectangles in the adjacent panels **(E,H,N,Q)**. Scale bars: 200 μm (original), 100 μm (magnified). Black arrowheads: granulation tissue; white arrowheads: subepidermal blister.

**Figure 3:**
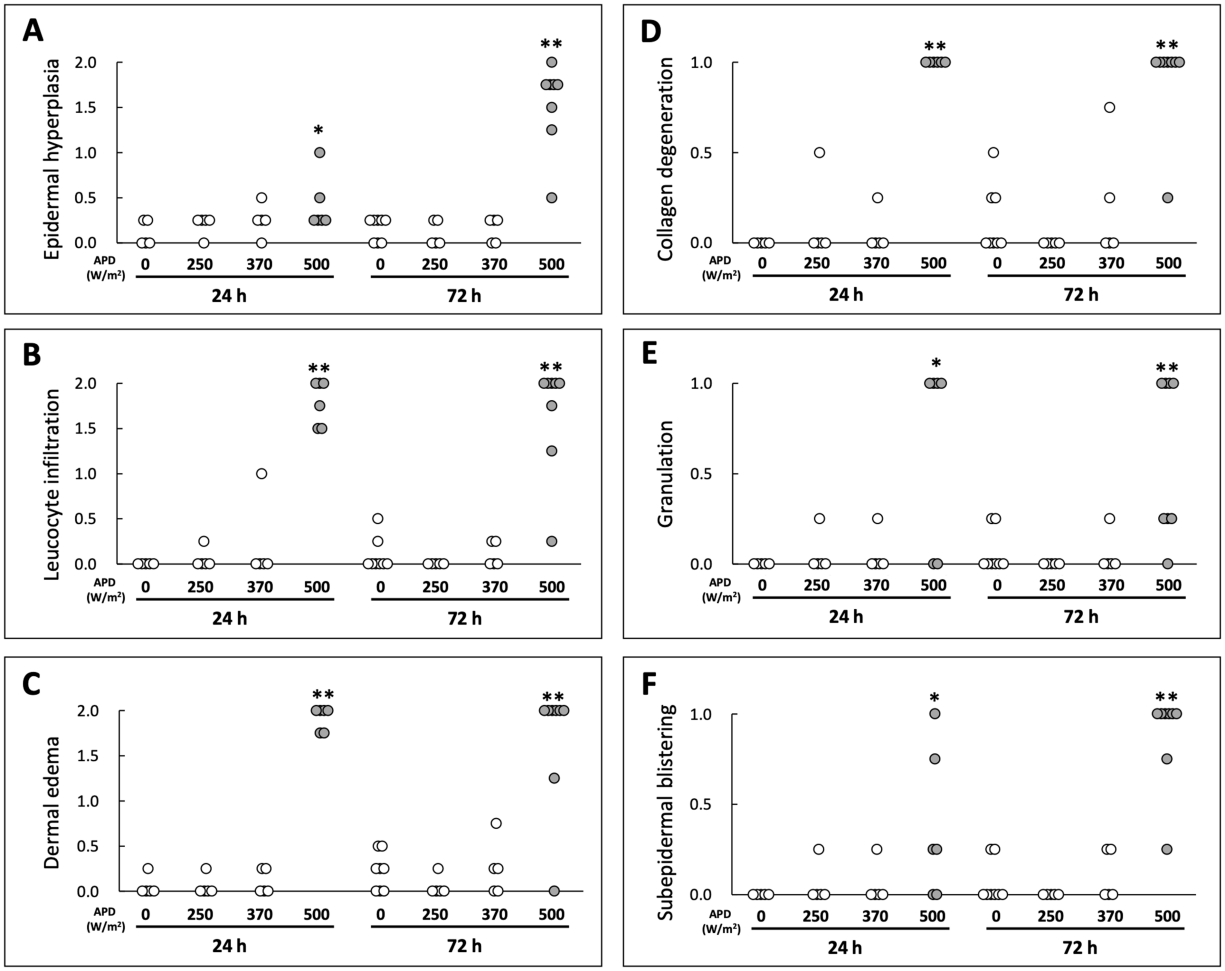
Scoring-based evaluation of histological changes following qMMW exposure. Scoring of skin damage at 24 and 72 h post-exposure for six parameters: **(A)** epidermal hyperplasia, **(B)** leukocyte infiltration, **(C)** dermal edema, **(D)** collagen degeneration, **(E)** granulation, **(F)** subepidermal blistering. Each dot represents the mean score of four independent evaluators per rat. **p < 0.01, *p < 0.05, *n* = 5–8. Details of the scoring method are provided in Materials and Methods.

H&E-stained cryosections were used to evaluate the histological changes in the skin tissue following qMMW exposure (Figures 2, 3). In the maximum exposure group (500 W/m^2^), the effects of MMW were observed at both 24 and 72 h post-exposure. At 24 h post-exposure, leukocyte infiltration (Figures 2E–J), dermal edema (Figures 2E,G,H,J), collagen degeneration (Figures 2G,J), granulation tissue formation (Figure 2F), subepidermal blister formation, and epidermal thickening were observed. At 72 h post-exposure, similar histological changes were detected (Figures 2N–S). Additionally, blister formation and epidermal thickening were more pronounced than at 24 h post-exposure (Figures 2O,R). Upon analyzing the scores of tissues, the 500 W/m^2^ exposure group showed significantly higher values for all six evaluated parameters than the sham group, both at 24 and 72 h post-exposure (Figure 3). However, no distinct morphological differences were observed among the sham, 250 W/m^2^, 370 W/m^2^, and cage control groups at either 24 h or 72 h post-exposure (Figures 2A–D,K–M). Moreover, no statistically significant differences were detected between the sham and low-exposure groups (Figure 3).

### 3.2 Expression of iNOS and Iba1 protein

To determine whether qMMW exposure induces inflammatory changes in the skin tissue, we analyzed the expression levels of iNOS and Iba1 proteins, which are both involved in inflammation.

**Figure 4:**
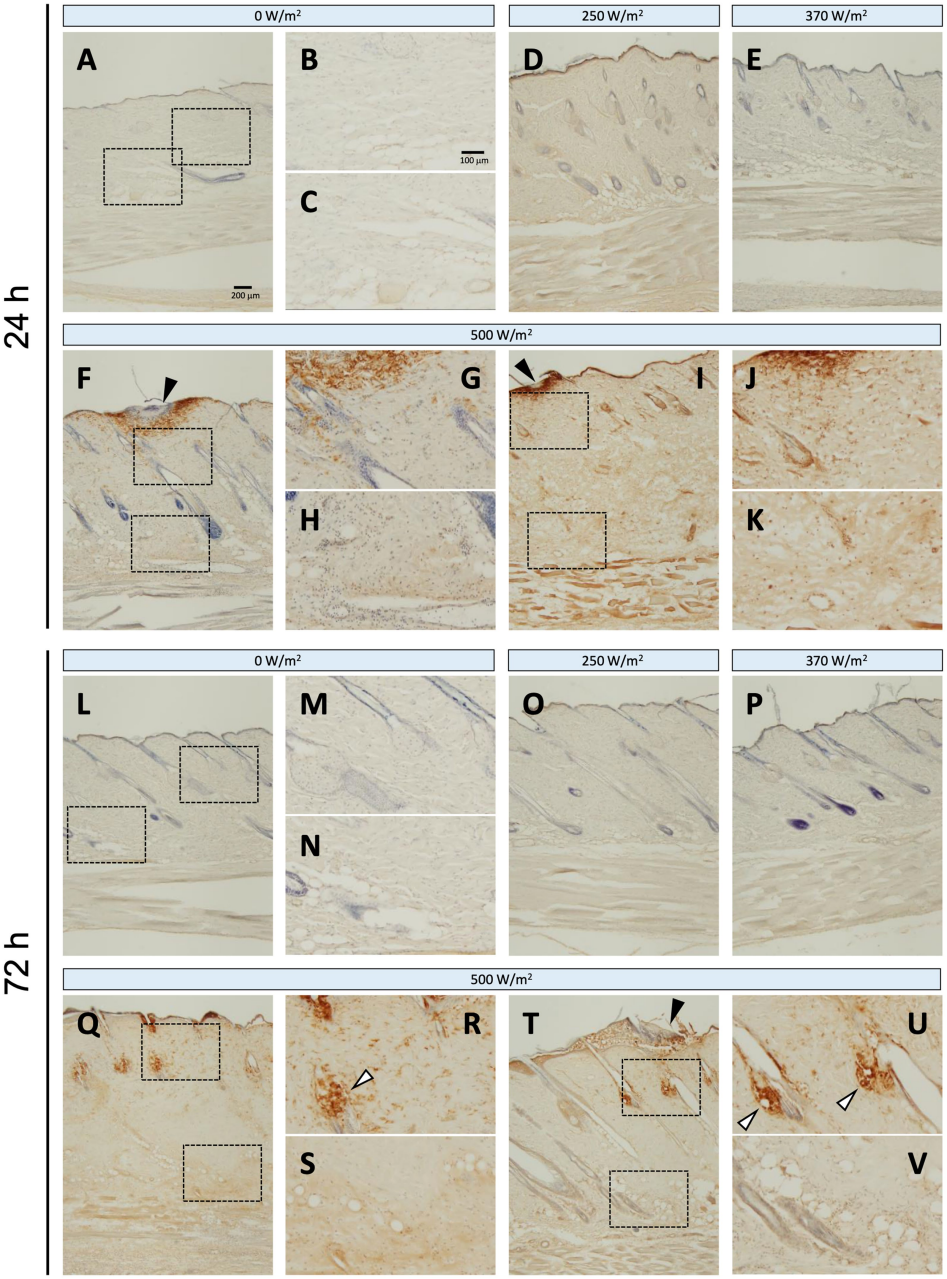
iNOS expression in rat skin after 26.5 GHz qMMW exposure. Immunostaining of iNOS at 24 h **(A–K)** and 72 h **(L–V)** post-exposure. Exposure groups: 0 W/m^2^ **(A–C,L–N)**, 250 W/m^2^ **(D,O)**, 370 W/m^2^ **(E,P)**, and 500 W/m^2^ **(F–K,Q–V)**. High-magnification images **(B,C,G,H,J,K,M,N,R,S,U,V)** show the dotted rectangles in adjacent left-side panels. Scale bars: 200 μm (original), 100 μm (magnified). Black arrowheads: the target site; white arrowheads: sebaceous glands; blue signal: hematoxylin counterstain.

Regarding iNOS protein expression, no positive signals were detected in the sham group at 24 or 72 h post-exposure (Figures 4A–C, L–N). In contrast, in the maximum exposure group (500 W/m^2^), the effects of exposure were observed at both 24 h and 72 h post-exposure. At 24 h post-exposure, positive signals were observed locally around the exposed area (Figures 4F,I). In addition, positive reactions were detected in the dermis and dWAT (Figures 4G,H,J,K). At 72 h post-exposure, similar positive signals were observed in the aforementioned regions (Figures 4Q–V). Furthermore, positive signals were detected in the sebaceous glands (Figures 4R,U). Conversely, no positive signals were detected in the 250 and 370 W/m^2^ exposure groups, similar to the sham group, at 24 and 72 h post-exposure (Figures 4D,E,O,P). Therefore, image analysis was not performed to determine iNOS protein expression.

**Figure 5:**
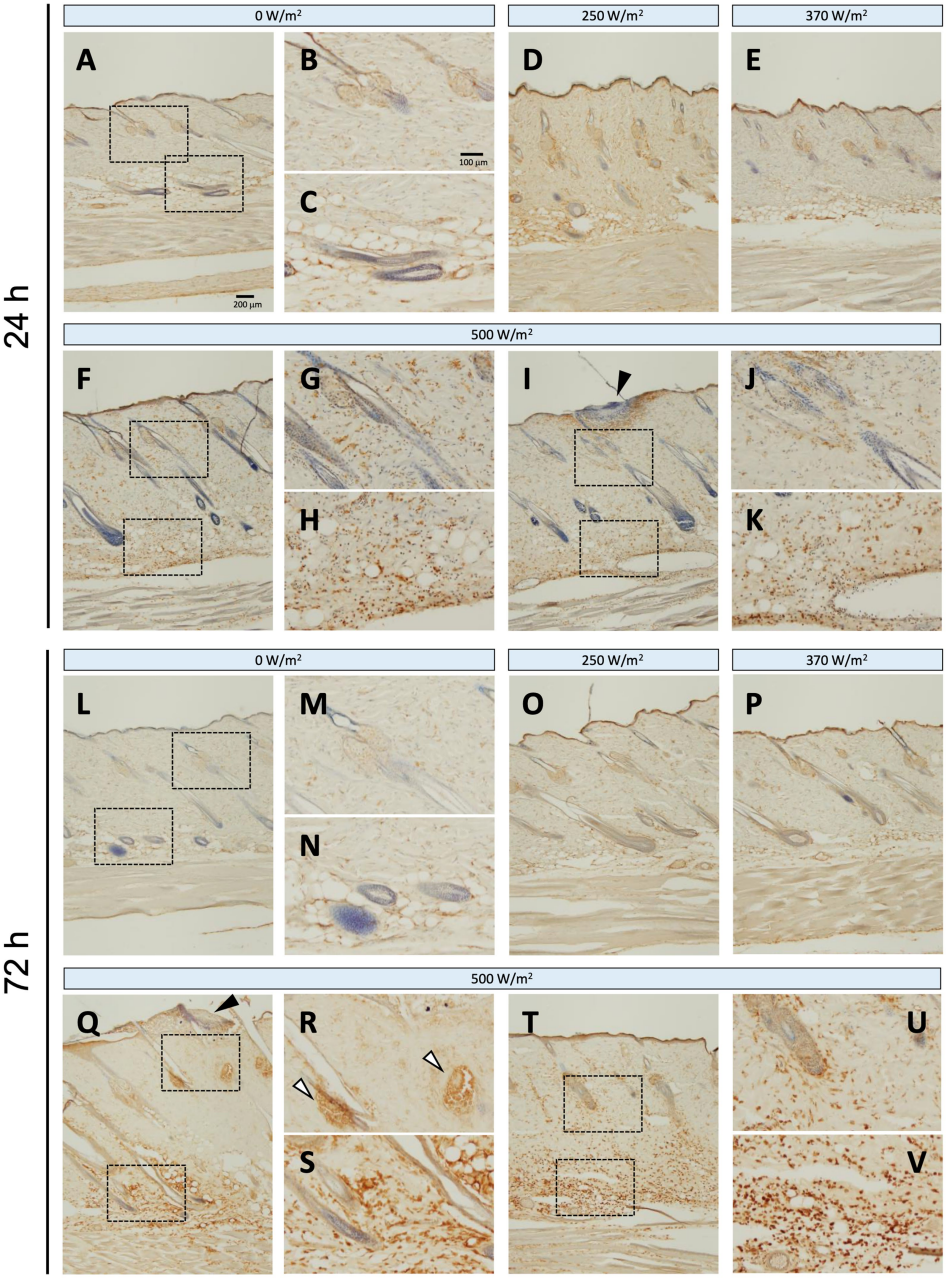
Increased Iba1 expression in rat skin after 26.5 GHz qMMW exposure. Immunostaining of Iba1 at 24 h **(A–K)** and 72 h **(L–V)** post-exposure. Exposure groups: 0 W/m^2^ **(A–C,L–N)**, 250 W/m^2^ **(D,O)**, 370 W/m^2^ **(E,P)**, 500 W/m^2^ **(F–K,Q–V)**. High-magnification images **(B,C,G,H,J,K,M,N,R,S,U,V)** show the dotted rectangles in adjacent left-side panels. Scale bars: 200 μm (original), 100 μm (magnified). Black arrowheads: the target site; white arrowheads: sebaceous glands; blue signal: hematoxylin counterstain.

Iba1 is constitutively expressed in the macrophages of intact skin (11); therefore, positive signals were observed in the dermis and dWAT across all the exposure groups (Figure 5). The number of Iba1-positive reactions tended to increase with increasing exposure intensity. In the maximum exposure group (500 W/m^2^), the effects of exposure were detected at 24 and 72 h post-exposure. At 24 h post-exposure, positive signals were detected in the dermis, dWAT, and around the exposed area (Figures 5F–K). At 72 h post-exposure, positive signals were observed in the sebaceous glands, in addition to the aforementioned regions (Figures 5Q–V). Although the 250 and 370 W/m^2^ exposure groups showed some effects of exposure, the signal intensity was lower than that of the 500 W/m^2^ exposure group (Figures 5D,E,O,P).

**Figure 6:**
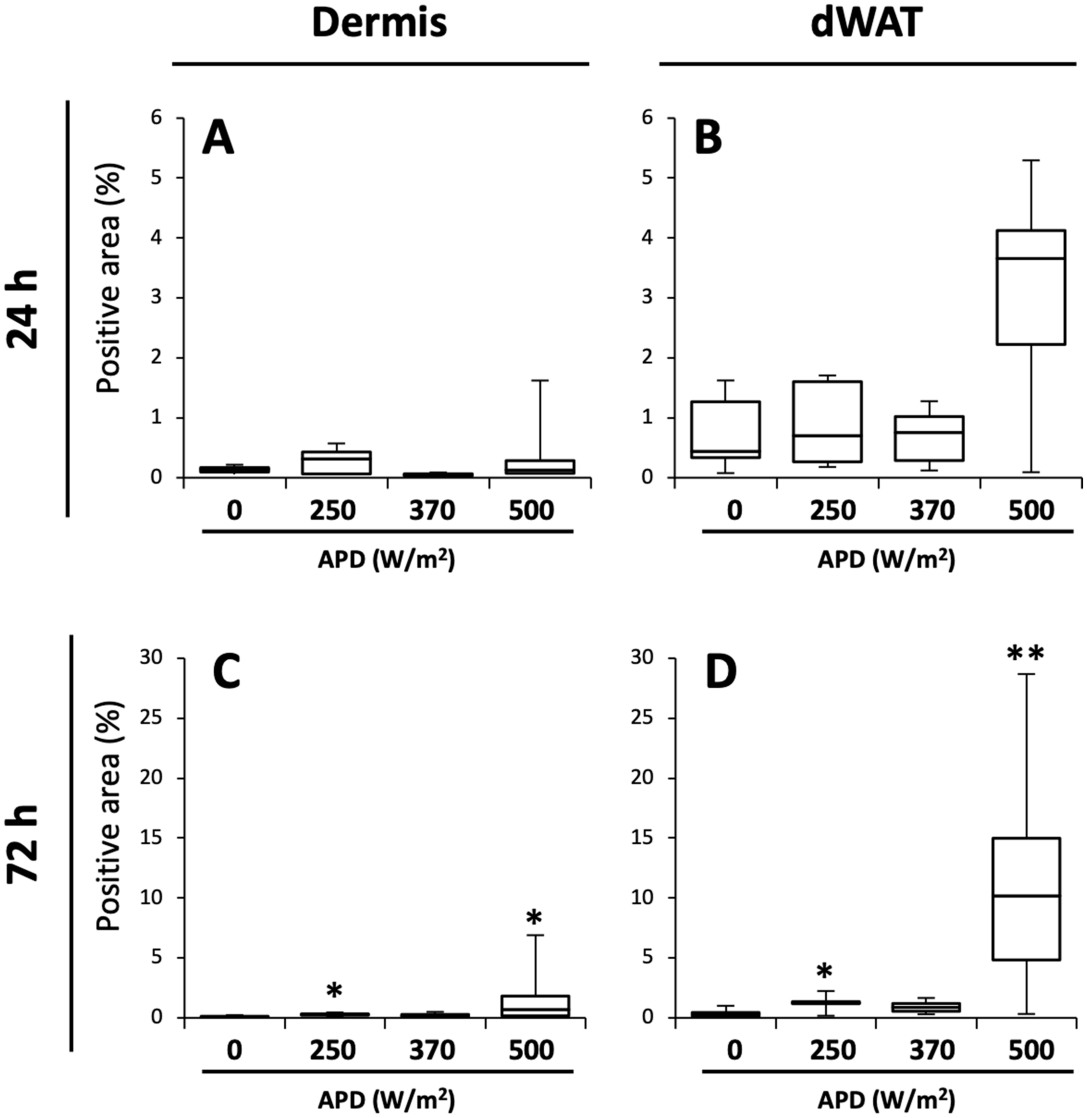
Quantification of Iba1-positive areas in the dermis and dWAT. Box plots of Iba1-positive area (%) in dermis **(A,C)** and dermal white adipose tissue (dWAT) **(B,D)** at 24 h **(A,B)** and 72 h **(C,D)** post-exposure. Median, interquartile ranges, and whiskers are shown. **p < 0.01, *p < 0.05, *n* = 5–8.

We quantified the Iba1-positive area per unit area and compared it with that of the sham group. Overall, more positive signals were detected in the dWAT than in the dermis (Figure 6). At 24 h post-exposure, some animals in the 500 W/m^2^ exposure group exhibited high values, although the differences were non-significant. At 72 h post-exposure, statistically significant differences in the dermis and dWAT were observed in 250 W/m^2^ and 500 W/m^2^ exposure groups (Figure 6).

### 3.3 Serum biomarker levels

**Figure 7:**
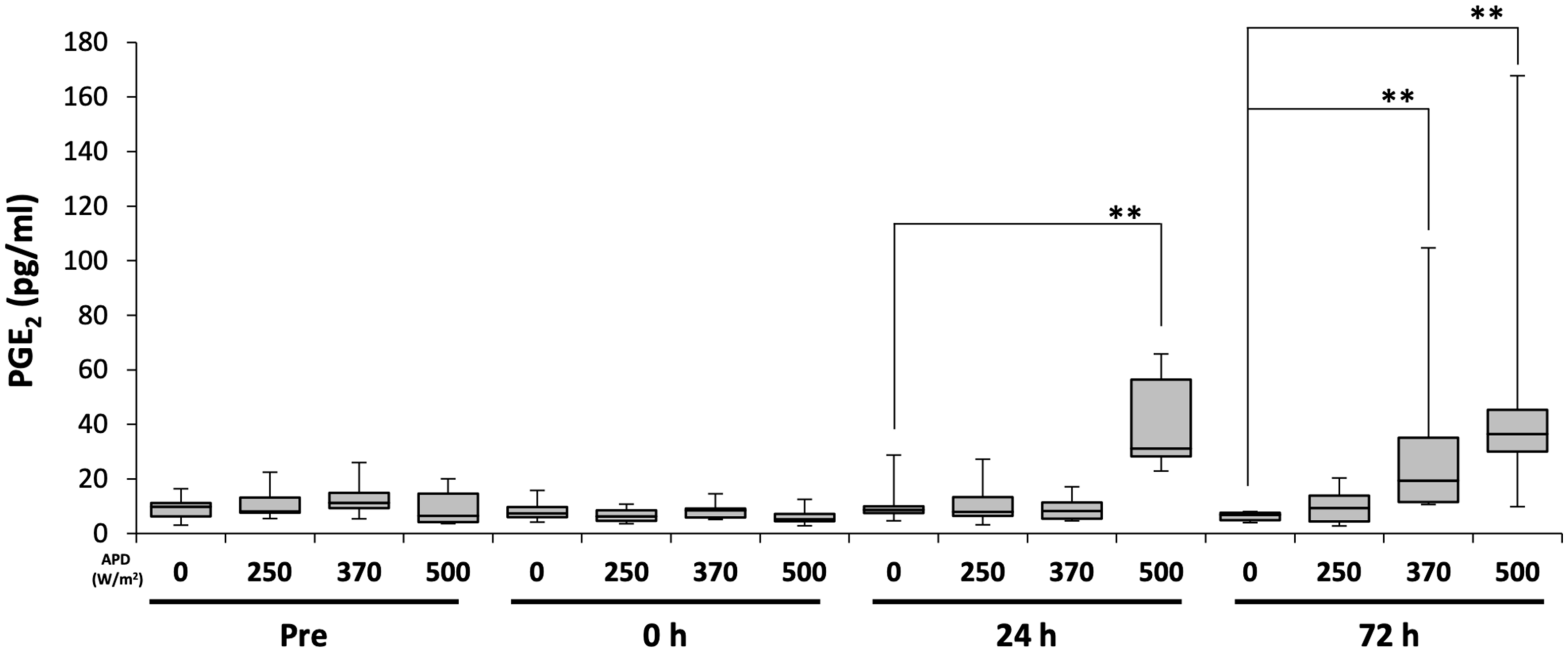
Changes in serum prostaglandin E_2_ (PGE_2_) levels following 26.5 GHz qMMW exposure. Serum PGE_2_ concentrations measured pre-exposure and at 0 h, 24 h, and 72 h post-exposure. **p < 0.01, *n* = 6–9.

To determine whether qMMW exposure can alter blood levels of multiple biomarkers, which are generated during tissue damage-induced inflammation, we measured biomarker concentrations at different time points. Considering PGE_2_, no intensity-dependent changes were observed immediately after exposure. However, 24 h post-exposure, a dose-dependent increase in PGE_2_ levels was observed. At 24 h post-exposure, the 500 W/m^2^ exposure group showed a significantly higher PGE_2_ level than the sham group. At 72 h post-exposure, both the 370 and 500 W/m^2^ exposure groups exhibited significantly higher PGE_2_ levels than the sham group (Figure 7). Conversely, TNF-*α* and IL-6 were undetectable at all time points (data not shown).

### 3.4 Temperature changes in the exposed skin tissue

The skin tissue temperature was measured during qMMW exposure to determine whether the observed biological changes could be attributed to increased tissue temperature. Table 1 presents the pre- and post-exposure skin temperatures at the irradiated site and the temperature increase after 18 min of exposure. Before exposure, the skin temperatures at the irradiation site were approximately 33–34°C across all exposure groups, with no significant differences detected between groups. By the end of the 18-min exposure, the skin temperatures reached approximately 44.8, 42.2, and 39.0°C in the 500, 370, and 250 W/m^2^ exposure groups, respectively. The corresponding temperature changes (ΔT) were 11.3 ± 0.9, 8.4 ± 0.7, and 5.6 ± 0.6°C (mean ± SD) in the 500, 370, and 250 W/m^2^ exposure groups, respectively. The time course of skin temperature changes at the target site during exposure has been reported in our previous study (8).

**Table 1:** Skin temperature changes during 26.5 GHz qMMW exposure.

APD (W/m^2^)	Pre (°C)	Post (°C)	ΔT (°C)	*N*
0	33.3 ± 0.8	33.1 ± 0.9	−0.2 ± 0.4	14
250	33.4 ± 0.5	39.0 ± 0.6	5.6 ± 0.6	10
370	33.9 ± 0.6	42.2 ± 0.9	8.4 ± 0.7	14
500	33.6 ± 0.7	44.8 ± 1.2	11.3 ± 0.9	15

The absolute skin temperature changes were measured in the target site exposed to qMMW before exposure (Pre) and at the end of exposure (Post). ΔT represents the temperature difference between pre- and post-exposure. All temperatures are expressed as mean ± SD for each exposure group.

## 4 Discussion

In this study, we analyzed the biological effects of localized exposure to 26.5 GHz qMMW on rat dorsal skin, focusing on histological changes and the expression of inflammation-related markers. The results revealed that the skin temperature at the exposure site increased to approximately 45°C at the highest exposure intensity (APD 500 W/m^2^), leading to histological changes in the epidermis, dermis, and dWAT, resembling those observed in thermal burns. Additionally, inflammation-related markers were significantly upregulated in the 500 W/m^2^ exposure group, showing a statistically significant increase compared with the sham group. However, because the skin temperature in this exposure condition reached 45°C, the observed effects could be due to thermal effects induced by qMMW exposure.

### 4.1 Burn-like tissue degeneration

The histological changes observed after qMMW exposure appeared to be associated with increased tissue temperature. It is well-known that heat exposure can induce histological alterations in skin tissues. For example, mouse skin exposed to a 90°C heated water bath for 7 s showed epidermal thickening (12). Furthermore, mouse skin exposed to a 54°C heated water bath for 25 s exhibited edema, collagen degeneration, and leukocyte infiltration in the dermis (13). Although the present study utilized qMMW exposure rather than a direct thermal load, similar histological changes were observed in the 500 W/m^2^ exposure group.

Epidermal thickening was observed in skin samples collected 3 days post-exposure, accompanied by subepidermal blisters, whereas edema, collagen degeneration, and leukocyte infiltration were detected in the dermis. Additionally, edema and leukocyte infiltration were observed in the dWAT. In contrast, no clear histological degeneration was observed in the 370 W/m^2^ exposure group (Figure 2). Direct temperature measurements obtained during exposure indicated that at APD 500 W/m^2^, the skin temperature exceeded 44°C in approximately 6 min and was maintained between 44 and 45°C until the end of the exposure period (Table 1) (8). In contrast, the skin temperature reached 42°C in the APD 370 W/m^2^ exposure group. These findings suggest that the observed histological changes could be attributed to the heat production induced by qMMW exposure at 500 W/m^2^, leading to burn-like tissue degeneration.

Similar results have been reported upon MMW exposure at different frequencies. Millenbaugh et al. observed histological changes, including neutrophil accumulation and collagen degeneration, following exposure to 35 GHz MMW (4). However, in their study, the skin temperature only reached 42°C. We hypothesized that this discrepancy may be due to differences in exposure duration. The severity of thermal injury depends on both the heat source temperature and the duration of exposure (14). Millenbaugh et al. utilized an exposure duration of 55 min, which is approximately three times longer than that in the present study (18 min). Therefore, although the skin temperature in the 370 W/m^2^ exposure group reached 42°C, the shorter exposure duration may have been insufficient to induce notable tissue damage. Additionally, differences in the MMW frequency and exposure methods may have contributed to the observed variations in biological effects.

### 4.2 Extended tissue effects of localized exposure

Histological changes induced by MMW exposure may extend deeper into the skin layers than anticipated previously. Generally, MMWs have short wavelengths, and their penetration into biological tissues is limited to a few millimeters (1). A human skin model simulation study estimated that qMMWs around 25 GHz penetrate to a depth of approximately 1 mm (15), suggesting that the 26.5 GHz qMMW used in this study would exhibit a similar penetration depth in rat skin. Based on the structural characteristics of the skin, a depth of 1 mm from the surface corresponds to the boundary between the dermis and dWAT (8, 9). Therefore, we initially expected histological changes induced by qMMW exposure to be confined to the dermis.

However, in our study, histological alterations were also observed in the dWAT, revealing that the effects of qMMW exposure extended deeper into the skin than predicted previously, beyond the theoretical penetration depth of the 26.5 GHz waves. Similar findings have been reported previously. For instance, in rat skin exposed to 35 GHz MMW, Millenbaugh et al. found that the effects extended to the dWAT, as well as to the underlying panniculus carnosus layer (4). Theoretically, 35 GHz MMW has a shorter wavelength than 26.5 GHz, implying that its penetration depth should be less than 1 mm. However, Millenbaugh et al. used older rats than those in the current study, making it unclear to what depth the 35 GHz waves actually penetrated. It is well known that with increasing age, the thickness of skin layers also increases (16), suggesting that it is unlikely that the 35 GHz waves penetrated beyond the dermis into deeper layers. Therefore, the effects of 26.5 GHz qMMW exposure at 500 W/m^2^ reaching deeper layers, such as the dWAT, may be primarily due to heat generation by qMMW energy in the skin, which subsequently propagated and induced these effects.

### 4.3 Inflammatory response in skin tissue

Localized inflammation-like responses were observed in skin exposed to 26.5 GHz qMMW at the maximum exposure intensity (Figure 3). In cases of skin injury, such as thermal burns, it is well-established that immune cells, including macrophages, leukocytes, and mast cells, interact with various inflammation-related factors, leading to a sequential inflammatory response (3, 17). The inflammatory process results in leukocyte infiltration, increased vascular permeability, and tissue destruction. Histological examination following qMMW exposure revealed leukocyte infiltration, collagen degeneration, and edema, suggesting that high-intensity qMMW exposure, which elevated the skin temperature to 45°C, may have induced the expression of inflammatory mediators.

Inflammatory mediators include free radicals such as reactive oxygen species and nitric oxide (NO), prostanoids such as prostaglandins and leukotrienes, and cytokines such as interleukins, TNF-*α*, and platelet-derived growth factor (18). In the current study, iNOS and Iba1 were used as inflammation-related markers to analyze inflammatory responses in the skin following qMMW exposure (Figures 4–6).

iNOS, an NO synthase, is induced in response to inflammation and stress, resulting in the production of NO, a free radical (19). NO regulates the progression of various stages of wound healing via leukocyte migration and cytokine production (19). In the current study, iNOS-positive cells were detected around the exposure site in the dermis and dWAT of the 500 W/m^2^ exposure group, suggesting that qMMW exposure induced an inflammatory response in the skin, leading to increased iNOS expression. This result is consistent with findings from rat burn models, where iNOS immunoreactivity was detected in keratinocytes, fibroblasts, endothelial cells, inflammatory cells, sweat glands, and hair follicles of dorsal skin exposed to heated soldering iron (20). These findings suggest that qMMW exposure induces iNOS expression and promotes the inflammatory response through local NO production. Furthermore, because iNOS-derived NO functions in wound healing (21), it may also contribute to tissue repair at the exposure site.

Iba1, another inflammation-related marker, is a monocyte lineage marker expressed in skin immune cells, including macrophages, dendritic cells, and Langerhans cells (11). These immune cells are activated in response to skin injury and play a role in promoting immune reactions. In the current study, Iba1-positive signals increased in the dermis and dWAT of the 500 W/m^2^ exposure group, suggesting that qMMW exposure activated immune cells in the skin (Figure 5). Additionally, activated macrophages produce additional inflammation-related factors, including iNOS (22), and the co-localized increase in iNOS and Iba1 expression observed in the same regions supports this finding.

The expression patterns of iNOS and Iba1 also gradually changed post-exposure, with notable expression in the sebaceous glands at 3 days post-exposure (Figures 4, 5). Sebaceous glands are involved in the regulation of lipid secretion and reportedly participate in immune responses (23, 24). Therefore, the post-exposure detection of iNOS and Iba1 expression in the sebaceous glands is an intriguing finding.

Image analysis of Iba1 expression showed a statistically significant increase even in the lower exposure groups (Figure 6). The skin temperatures at 18 min post-exposure reached ~39°C in the 250 W/m^2^ group (Table 1). Although this temperature was insufficient to induce thermal burns or tissue damage, it may have subjected immune cells in the skin to thermal stress, leading to a slight activation. However, as demonstrated in Figure 5, the increase in Iba1 expression in the lower exposure groups was markedly smaller than in the 500 W/m^2^ group, suggesting that it is unlikely to progress to a notable inflammation-like response.

### 4.4 Systemic reaction after localized qMMW exposure

PGE_2_, induced by 26.5 GHz qMMW exposure, is an inflammatory mediator belonging to prostanoids that is generated during inflammation and synthesized from arachidonic acid in the cell membrane upon tissue damage (25). Locally produced PGE_2_ not only activates mast cells and induces the expression of inflammatory cytokines but also promotes histamine release through mast cell degranulation (25–27). Released histamine increases vascular permeability, leading to the development of edema (26). In the current study, edema was observed in the dermis and dWAT of the 500 W/m^2^ exposure group, suggesting that qMMW exposure could trigger a series of PGE_2_-related inflammation-like responses (Figure 7).

Furthermore, in the 500 W/m^2^ exposure group, serum PGE_2_ levels increased from 24 h post-exposure, likely reflecting the progression of the inflammatory response due to PGE_2_ induction. Previous studies using burn injury rat models have also reported increased PGE_2_ levels in body fluids, accompanied by epidermal thickening and edema as inflammatory responses in the skin (28), consistent with our findings. Additionally, according to Okayama et al., local skin burns were found to affect the central nervous system, resulting in increased PGE_2_ concentrations in the cerebrospinal fluid and activation of PGE_2_ receptors in the central nervous system (29). These findings support the notion that the increase in serum PGE_2_ levels following qMMW exposure indicates tissue damage and subsequent inflammatory response. Moreover, elevated serum PGE_2_ levels suggest that a localized inflammatory response may have systemic effects, highlighting the need for further studies to investigate its potential impact on the central nervous system and other organs.

In contrast, in the exposure group at 370 W/m^2^, serum PGE_2_ levels significantly increased 72 h post-exposure (Figure 7) despite the absence of apparent tissue damage (Figures 2, 3). PGE_2_ is reportedly produced by keratinocytes in the skin and fibroblasts in the dermis in response to thermal stress (30, 31). Additionally, the temperature-sensitive receptors TRPV3 and TRPM4 are expressed in keratinocytes (32, 33). TRPV3 is activated within a physiological temperature range of 33–39°C, triggering the secretion of PGE_2_ and NO (21). TRPM4 is activated by warm temperatures of 15–35°C and is known to be involved in modulating the immune responses (33, 34). These molecules are implicated in conveying temperature-related information (21). The increased PGE_2_ level may not be associated with an inflammatory response but rather serves as part of a homeostatic mechanism for thermoregulation and blood flow control. Although skin temperature in the 370 W/m^2^ exposure group did not reach levels sufficient to induce burns, it was likely sufficient to activate these TRP channels. Therefore, the increase in PGE_2_ observed in the 370 W/m^2^ exposure group could be a physiological response to environmental temperature changes for homeostasis maintenance rather than a sign of inflammation.

The cytokines TNF-*α* and IL-6, which act as inflammatory mediators, play central roles in inflammatory responses (35). Therefore, if these molecules were induced by qMMW exposure, it would provide strong evidence that exposure to 26.5 GHz qMMW could trigger an inflammatory response. However, in the current study, both TNF-α and IL-6 were undetectable, and we could not obtain direct evidence that qMMW exposure induces inflammation. One possible explanation for the absence of detectable cytokines in the bloodstream is that the extent and severity of the burn area caused by the exposure were relatively small. Even at the highest exposure intensity used in this study, the burn-like response was confined to an area of approximately 1 cm^2^, corresponding to ~0.2% of the total body surface area, as estimated using Meeh’s formula (36, 37). A 20% burn area in rat burn models was associated with elevated blood cytokine levels (38). Therefore, a burn area of only 0.2% may be insufficient to induce detectable cytokine levels in the bloodstream. Nevertheless, other studies using mouse burn models detected no increase in blood cytokine levels even when the burn area reached 20% of the total body surface area (28). These findings suggest that cytokine detectability in the bloodstream may vary depending on the experimental conditions in rodent burn models. Thus, in future studies, implementing a more sensitive detection system may facilitate the determination of whether qMMW exposure influences cytokine expression.

### 4.5 Threshold of exposure intensity for the skin effects

In the current study, the threshold for qMMW exposure-induced inflammation was predicted to range between 370 and 500 W/m^2^ APD. Upon histological analysis using H&E staining, substantial tissue changes, which served as an indicator of the inflammatory response, were observed only in the 500 W/m^2^ exposure group (Figures 2, 3). Analysis of inflammation-related markers revealed a statistically significant increase in the 500 W/m^2^ exposure group, suggesting that the heat generated by high-intensity qMMW exposure induces an inflammatory response in the tissue. Some inflammation-related markers also showed responses in the lower exposure groups, although these were considered adaptive responses for homeostasis maintenance rather than true inflammatory reactions, likely resulting from mild thermal stress in tissues and cells. Therefore, under the conditions of this study, the threshold for 26.5 GHz qMMW-induced inflammation was estimated to range between 370 and 500 W/m^2^ APD.

The International Commission for Non-Ionizing Radiation Protection (ICNIRP) guidelines state that high-frequency electromagnetic wave exposure that increases the local temperature of human organs and tissues beyond 41°C is potentially harmful (6). The estimated exposure level that did not exceed this threshold is an APD of 200 W/m^2^. Accordingly, the guideline values were set at 100 W/m^2^ for occupational exposure (with a safety factor of 1/2) and 20 W/m^2^ for general public exposure (with a safety factor of 1/10). The threshold values obtained in this study were derived from rat skin and cannot be directly extrapolated to humans. However, the occupational exposure guideline value was less than one-fourth of the estimated 370–500 W/m^2^ threshold. Furthermore, it is highly unlikely that inflammatory responses in skin tissues will occur at this guideline level. Therefore, current guidelines appear to ensure the safety of human organs and tissues. Nevertheless, future studies should continue safety evaluations considering different frequencies, repeated exposure at lower intensities, and variations among various tissue types.

## 5 Conclusion

In this study, the dorsal skin of rats was exposed to localized 26.5 GHz qMMW, and the resulting histological changes and expression of inflammation-related molecules were analyzed. Histological changes and inflammation-like responses were observed only at an exposure intensity of 500 W/m^2^. Few studies have demonstrated inflammation-like responses in the skin due to MMW exposure, and to the best of our knowledge, this study is the first to clearly define the threshold using APD as a reference. These findings may contribute useful evidence for future revisions of exposure guidelines.

## Data Availability

The raw data supporting the conclusions of this article will be made available by the authors, without undue reservation.
